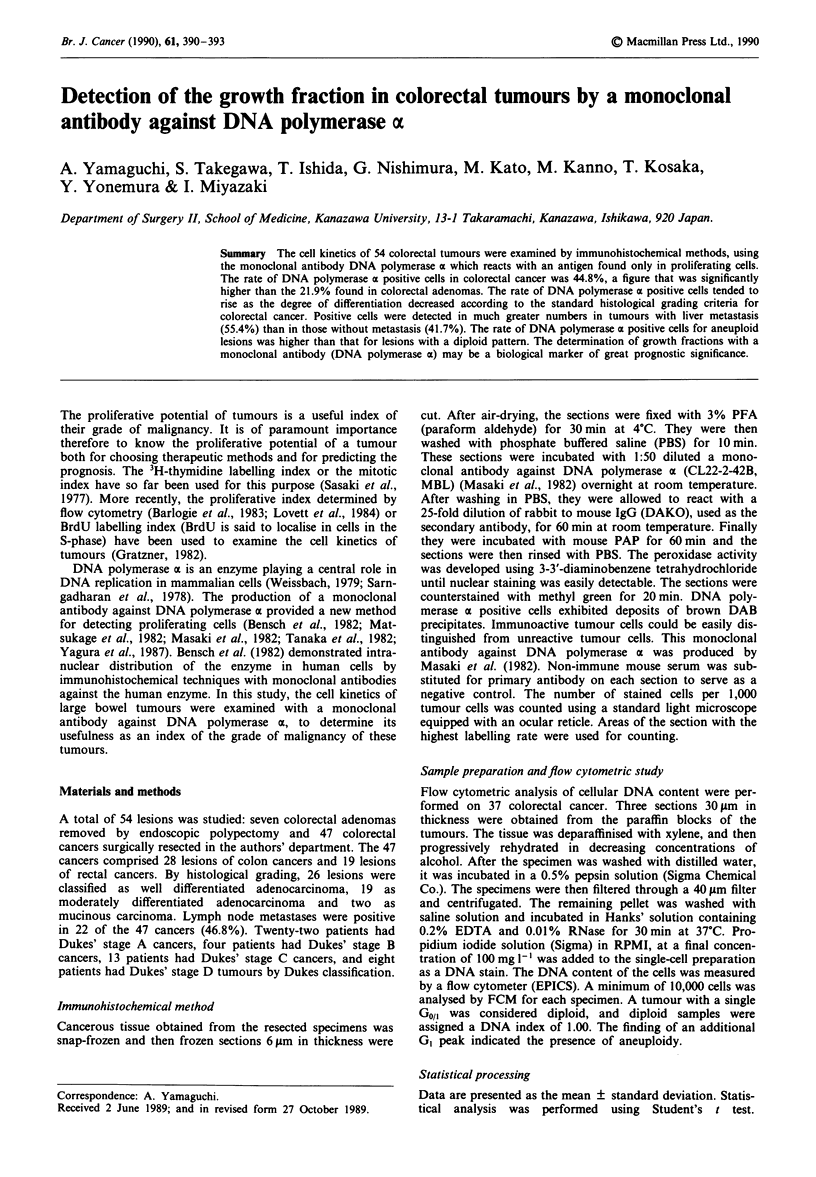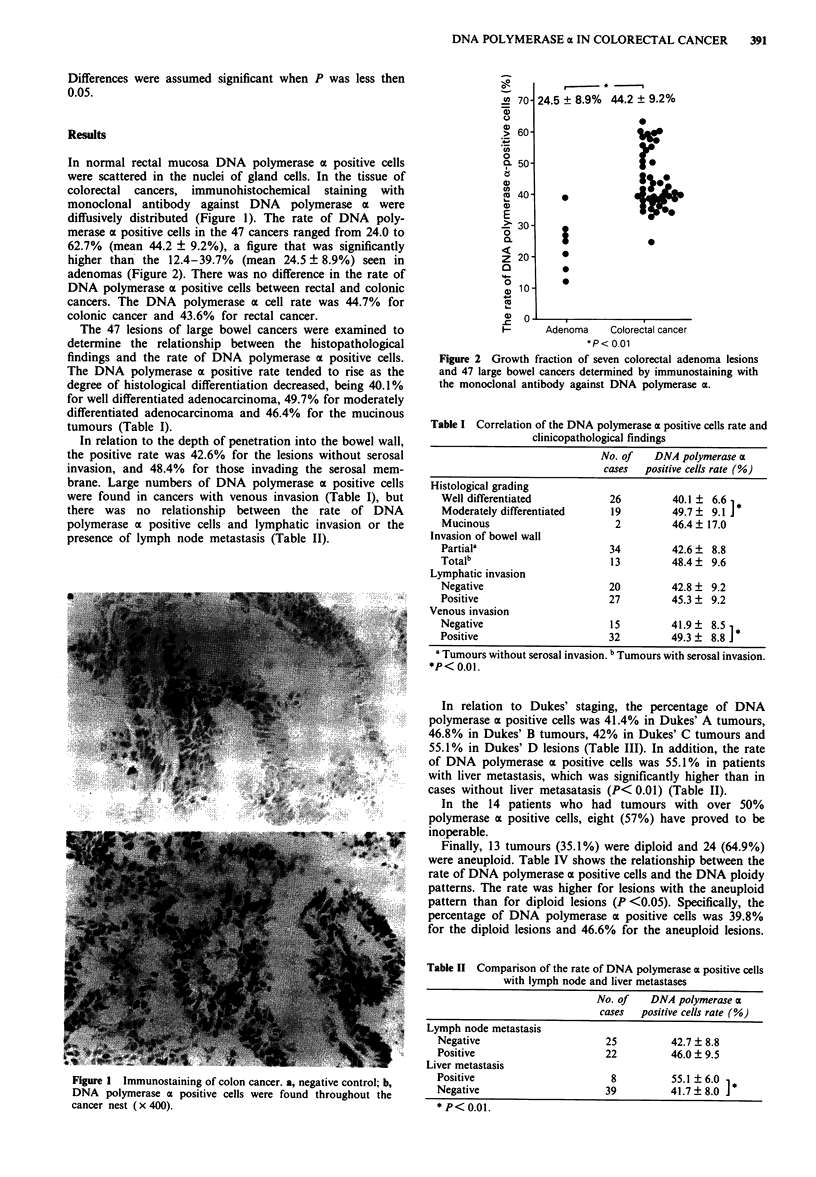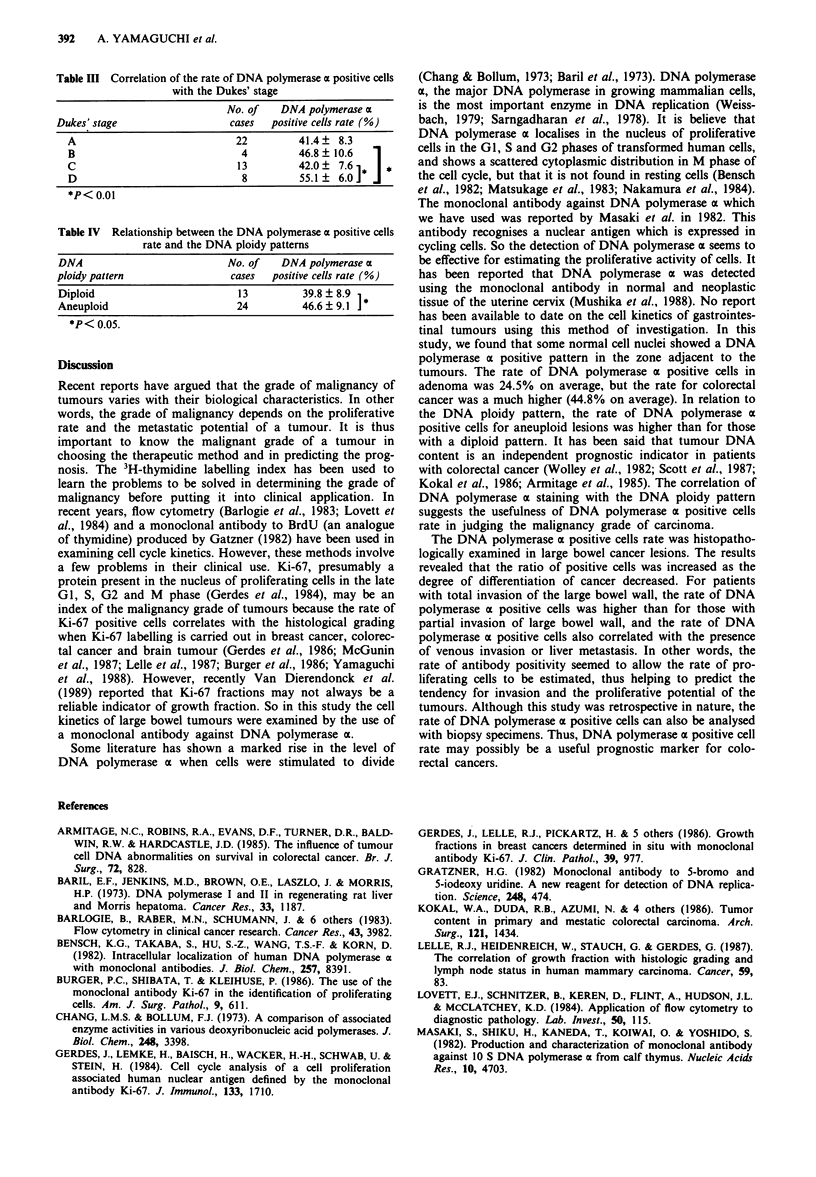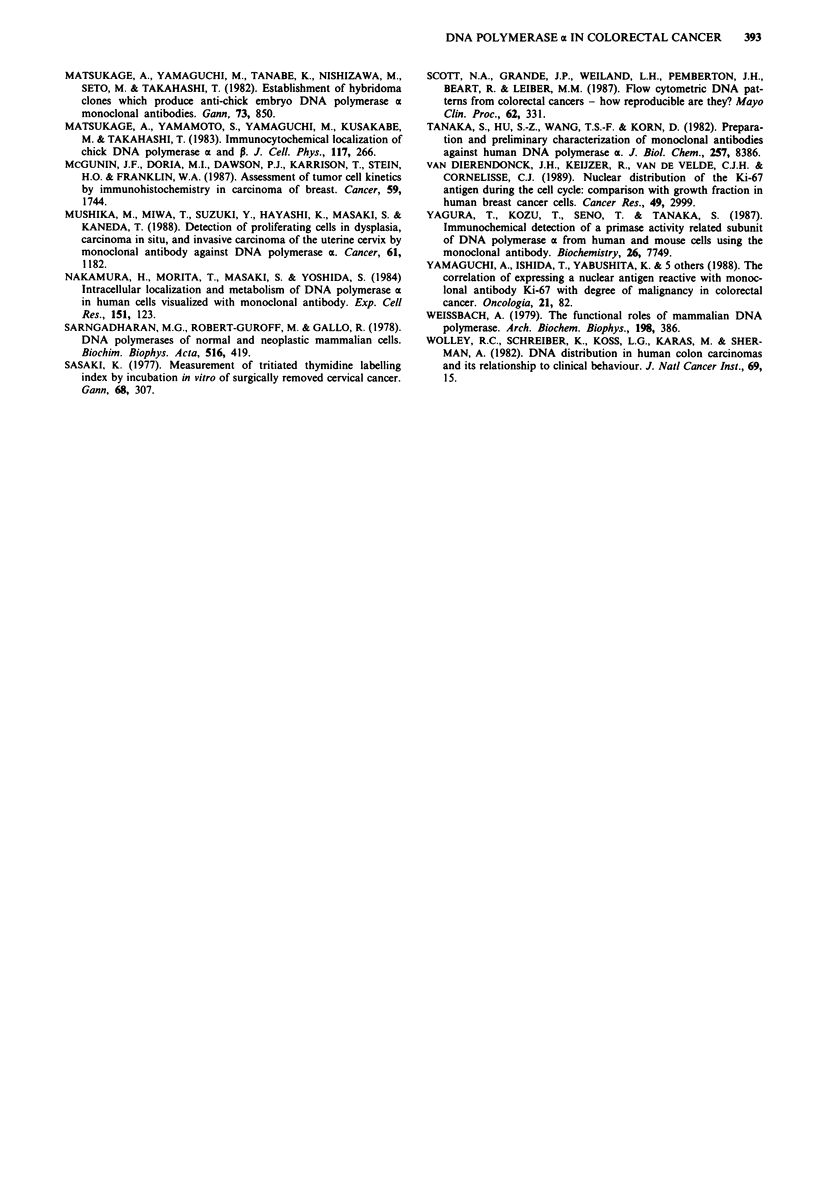# Detection of the growth fraction in colorectal tumours by a monoclonal antibody against DNA polymerase alpha.

**DOI:** 10.1038/bjc.1990.85

**Published:** 1990-03

**Authors:** A. Yamaguchi, S. Takegawa, T. Ishida, G. Nishimura, M. Kato, M. Kanno, T. Kosaka, Y. Yonemura, I. Miyazaki

**Affiliations:** Department of Surgery II, School of Medicine, Kanazawa University, Ishikawa, Japan.

## Abstract

**Images:**


Br. J. Cancer (1990), 61, 390-393 © Macmillan Press Ltd., 1990

### Detection of the growth fraction in colorectal tumours by a monoclonal antibody against DNA polymerase α

A. Yamaguchi, S. Takegawa, T. Ishida, G. Nishimura, M. Kato, M. Kanno, T. Kosaka, Y. Yonemura & I. Miyazaki

*Department of Surgery II, School of Medicine, Kanazawa University, 13-1 Takaramachi, Kanazawa, Ishikawa, 920 Japan.*

**Summary** The cell kinetics of 54 colorectal tumours were examined by immunohistochemical methods, using the monoclonal antibody DNA polymerase α which reacts with an antigen found only in proliferating cells. The rate of DNA polymerase α positive cells in colorectal cancer was 44.8%, a figure that was significantly higher than the 21.9% found in colorectal adenomas. The rate of DNA polymerase α positive cells tended to rise as the degree of differentiation decreased according to the standard histological grading criteria for colorectal cancer. Positive cells were detected in much greater numbers in tumours with liver metastasis (55.4%) than in those without metastasis (41.7%). The rate of DNA polymerase α positive cells for aneuploid lesions was higher than that for lesions with a diploid pattern. The determination of growth fractions with a monoclonal antibody (DNA polymerase α) may be a biological marker of great prognostic significance.

The proliferative potential of tumours is a useful index of their grade of malignancy. It is of paramount importance therefore to know the proliferative potential of a tumour both for choosing therapeutic methods and for predicting the prognosis. The $^3$H-thymidine labelling index or the mitotic index have so far been used for this purpose (Sasaki *et al.*, 1977). More recently, the proliferative index determined by flow cytometry (Barlogie *et al.*, 1983; Lovett *et al.*, 1984) or BrdU labelling index (BrdU is said to localise in cells in the S-phase) have been used to examine the cell kinetics of tumours (Gratzner, 1982).

DNA polymerase α is an enzyme playing a central role in DNA replication in mammalian cells (Weissbach, 1979; Sarngadharan *et al.*, 1978). The production of a monoclonal antibody against DNA polymerase α provided a new method for detecting proliferating cells (Bensch *et al.*, 1982; Matsukage *et al.*, 1982; Masaki *et al.*, 1982; Tanaka *et al.*, 1982; Yagura *et al.*, 1987). Bensch *et al.* (1982) demonstrated intranuclear distribution of the enzyme in human cells by immunohistochemical techniques with monoclonal antibodies against the human enzyme. In this study, the cell kinetics of large bowel tumours were examined with a monoclonal antibody against DNA polymerase α, to determine its usefulness as an index of the grade of malignancy of these tumours.

### Materials and methods

A total of 54 lesions was studied: seven colorectal adenomas removed by endoscopic polypectomy and 47 colorectal cancers surgically resected in the authors' department. The 47 cancers comprised 28 lesions of colon cancers and 19 lesions of rectal cancers. By histological grading, 26 lesions were classified as well differentiated adenocarcinoma, 19 as moderately differentiated adenocarcinoma and two as mucinous carcinoma. Lymph node metastases were positive in 22 of the 47 cancers (46.8%). Twenty-two patients had Dukes' stage A cancers, four patients had Dukes' stage B cancers, 13 patients had Dukes' stage C cancers, and eight patients had Dukes' stage D tumours by Dukes classification.

### *Immunohistochemical method*

Cancerous tissue obtained from the resected specimens was snap-frozen and then frozen sections 6 μm in thickness were cut. After air-drying, the sections were fixed with 3% PFA (paraform aldehyde) for 30 min at 4°C. They were then washed with phosphate buffered saline (PBS) for 10 min. These sections were incubated with 1:50 diluted a monoclonal antibody against DNA polymerase α (CL22-2-42B, MBL) (Masaki *et al.*, 1982) overnight at room temperature. After washing in PBS, they were allowed to react with a 25-fold dilution of rabbit to mouse IgG (DAKO), used as the secondary antibody, for 60 min at room temperature. Finally they were incubated with mouse PAP for 60 min and the sections were then rinsed with PBS. The peroxidase activity was developed using 3-3'-diaminobenzene tetrahydrochloride until nuclear staining was easily detectable. The sections were counterstained with methyl green for 20 min. DNA polymerase α positive cells exhibited deposits of brown DAB precipitates. Immunoactive tumour cells could be easily distinguished from unreactive tumour cells. This monoclonal antibody against DNA polymerase α was produced by Masaki *et al.* (1982). Non-immune mouse serum was substituted for primary antibody on each section to serve as a negative control. The number of stained cells per 1,000 tumour cells was counted using a standard light microscope equipped with an ocular reticle. Areas of the section with the highest labelling rate were used for counting.

### *Sample preparation and flow cytometric study*

Flow cytometric analysis of cellular DNA content were performed on 37 colorectal cancer. Three sections 30 μm in thickness were obtained from the paraffin blocks of the tumours. The tissue was deparaffinised with xylene, and then progressively rehydrated in decreasing concentrations of alcohol. After the specimen was washed with distilled water, it was incubated in a 0.5% pepsin solution (Sigma Chemical Co.). The specimens were then filtered through a 40 μm filter and centrifugated. The remaining pellet was washed with saline solution and incubated in Hanks' solution containing 0.2% EDTA and 0.01% RNase for 30 min at 37°C. Propidium iodide solution (Sigma) in RPMI, at a final concentration of 100 mg $l^{-1}$ was added to the single-cell preparation as a DNA stain. The DNA content of the cells was measured by a flow cytometer (EPICS). A minimum of 10,000 cells was analysed by FCM for each specimen. A tumour with a single $G_{0/1}$ was considered diploid, and diploid samples were assigned a DNA index of 1.00. The finding of an additional $G_1$ peak indicated the presence of aneuploidy.

### *Statistical processing*

Data are presented as the mean ± standard deviation. Statistical analysis was performed using Student's *t* test.

Correspondence: A. Yamaguchi.
Received 2 June 1989; and in revised form 27 October 1989.

Differences were assumed significant when $P$ was less then 0.05.

### Results

In normal rectal mucosa DNA polymerase α positive cells were scattered in the nuclei of gland cells. In the tissue of colorectal cancers, immunohistochemical staining with monoclonal antibody against DNA polymerase α were diffusively distributed (Figure 1). The rate of DNA polymerase α positive cells in the 47 cancers ranged from 24.0 to 62.7% (mean 44.2 ± 9.2%), a figure that was significantly higher than the 12.4–39.7% (mean 24.5 ± 8.9%) seen in adenomas (Figure 2). There was no difference in the rate of DNA polymerase α positive cells between rectal and colonic cancers. The DNA polymerase α cell rate was 44.7% for colonic cancer and 43.6% for rectal cancer.

The 47 lesions of large bowel cancers were examined to determine the relationship between the histopathological findings and the rate of DNA polymerase α positive cells. The DNA polymerase α positive rate tended to rise as the degree of histological differentiation decreased, being 40.1% for well differentiated adenocarcinoma, 49.7% for moderately differentiated adenocarcinoma and 46.4% for the mucinous tumours (Table I).

In relation to the depth of penetration into the bowel wall, the positive rate was 42.6% for the lesions without serosal invasion, and 48.4% for those invading the serosal membrane. Large numbers of DNA polymerase α positive cells were found in cancers with venous invasion (Table I), but there was no relationship between the rate of DNA polymerase α positive cells and lymphatic invasion or the presence of lymph node metastasis (Table II).

**Figure 1** Immunostaining of colon cancer. **a**, negative control; **b**, DNA polymerase α positive cells were found throughout the cancer nest (× 400).

**Figure 2** Growth fraction of seven colorectal adenoma lesions and 47 large bowel cancers determined by immunostaining with the monoclonal antibody against DNA polymerase α.

**Table I** Correlation of the DNA polymerase α positive cells rate and clinicopathological findings

| | *No. of cases* | *DNA polymerase α positive cells rate (%)* |
|---|---|---|
| Histological grading | | |
| Well differentiated | 26 | 40.1 ± 6.6 ]* |
| Moderately differentiated | 19 | 49.7 ± 9.1 ]* |
| Mucinous | 2 | 46.4 ± 17.0 |
| Invasion of bowel wall | | |
| Partial[a] | 34 | 42.6 ± 8.8 |
| Total[b] | 13 | 48.4 ± 9.6 |
| Lymphatic invasion | | |
| Negative | 20 | 42.8 ± 9.2 |
| Positive | 27 | 45.3 ± 9.2 |
| Venous invasion | | |
| Negative | 15 | 41.9 ± 8.5 ]* |
| Positive | 32 | 49.3 ± 8.8 ]* |

[a] Tumours without serosal invasion. [b] Tumours with serosal invasion. *$P < 0.01$.

In relation to Dukes' staging, the percentage of DNA polymerase α positive cells was 41.4% in Dukes' A tumours, 46.8% in Dukes' B tumours, 42% in Dukes' C tumours and 55.1% in Dukes' D lesions (Table III). In addition, the rate of DNA polymerase α positive cells was 55.1% in patients with liver metastasis, which was significantly higher than in cases without liver metasatasis ($P < 0.01$) (Table II).

In the 14 patients who had tumours with over 50% polymerase α positive cells, eight (57%) have proved to be inoperable.

Finally, 13 tumours (35.1%) were diploid and 24 (64.9%) were aneuploid. Table IV shows the relationship between the rate of DNA polymerase α positive cells and the DNA ploidy patterns. The rate was higher for lesions with the aneuploid pattern than for diploid lesions ($P < 0.05$). Specifically, the percentage of DNA polymerase α positive cells was 39.8% for the diploid lesions and 46.6% for the aneuploid lesions.

**Table II** Comparison of the rate of DNA polymerase α positive cells with lymph node and liver metastases

| | *No. of cases* | *DNA polymerase α positive cells rate (%)* |
|---|---|---|
| Lymph node metastasis | | |
| Negative | 25 | 42.7 ± 8.8 |
| Positive | 22 | 46.0 ± 9.5 |
| Liver metastasis | | |
| Positive | 8 | 55.1 ± 6.0 ]* |
| Negative | 39 | 41.7 ± 8.0 ]* |

* $P < 0.01$.

**Table III** Correlation of the rate of DNA polymerase α positive cells with the Dukes' stage

| *Dukes' stage* | *No. of cases* | *DNA polymerase α positive cells rate (%)* |
|---|---|---|
| A | 22 | 41.4 ± 8.3 |
| B | 4 | 46.8 ± 10.6 |
| C | 13 | 42.0 ± 7.6 |
| D | 8 | 55.1 ± 6.0 |

*$P < 0.01$

**Table IV** Relationship between the DNA polymerase α positive cells rate and the DNA ploidy patterns

| *DNA ploidy pattern* | *No. of cases* | *DNA polymerase α positive cells rate (%)* |
|---|---|---|
| Diploid | 13 | 39.8 ± 8.9 |
| Aneuploid | 24 | 46.6 ± 9.1 |

*$P < 0.05$.

### Discussion

Recent reports have argued that the grade of malignancy of tumours varies with their biological characteristics. In other words, the grade of malignancy depends on the proliferative rate and the metastatic potential of a tumour. It is thus important to know the malignant grade of a tumour in choosing the therapeutic method and in predicting the prognosis. The $^3$H-thymidine labelling index has been used to learn the problems to be solved in determining the grade of malignancy before putting it into clinical application. In recent years, flow cytometry (Barlogie *et al.*, 1983; Lovett *et al.*, 1984) and a monoclonal antibody to BrdU (an analogue of thymidine) produced by Gatzner (1982) have been used in examining cell cycle kinetics. However, these methods involve a few problems in their clinical use. Ki-67, presumably a protein present in the nucleus of proliferating cells in the late G1, S, G2 and M phase (Gerdes *et al.*, 1984), may be an index of the malignancy grade of tumours because the rate of Ki-67 positive cells correlates with the histological grading when Ki-67 labelling is carried out in breast cancer, colorectal cancer and brain tumour (Gerdes *et al.*, 1986; McGunin *et al.*, 1987; Lelle *et al.*, 1987; Burger *et al.*, 1986; Yamaguchi *et al.*, 1988). However, recently Van Dierendonck *et al.* (1989) reported that Ki-67 fractions may not always be a reliable indicator of growth fraction. So in this study the cell kinetics of large bowel tumours were examined by the use of a monoclonal antibody against DNA polymerase α.

Some literature has shown a marked rise in the level of DNA polymerase α when cells were stimulated to divide (Chang & Bollum, 1973; Baril *et al.*, 1973). DNA polymerase α, the major DNA polymerase in growing mammalian cells, is the most important enzyme in DNA replication (Weissbach, 1979; Sarngadharan *et al.*, 1978). It is believe that DNA polymerase α localises in the nucleus of proliferative cells in the G1, S and G2 phases of transformed human cells, and shows a scattered cytoplasmic distribution in M phase of the cell cycle, but that it is not found in resting cells (Bensch *et al.*, 1982; Matsukage *et al.*, 1983; Nakamura *et al.*, 1984). The monoclonal antibody against DNA polymerase α which we have used was reported by Masaki *et al.* in 1982. This antibody recognises a nuclear antigen which is expressed in cycling cells. So the detection of DNA polymerase α seems to be effective for estimating the proliferative activity of cells. It has been reported that DNA polymerase α was detected using the monoclonal antibody in normal and neoplastic tissue of the uterine cervix (Mushika *et al.*, 1988). No report has been available to date on the cell kinetics of gastrointestinal tumours using this method of investigation. In this study, we found that some normal cell nuclei showed a DNA polymerase α positive pattern in the zone adjacent to the tumours. The rate of DNA polymerase α positive cells in adenoma was 24.5% on average, but the rate for colorectal cancer was a much higher (44.8% on average). In relation to the DNA ploidy pattern, the rate of DNA polymerase α positive cells for aneuploid lesions was higher than for those with a diploid pattern. It has been said that tumour DNA content is an independent prognostic indicator in patients with colorectal cancer (Wolley *et al.*, 1982; Scott *et al.*, 1987; Kokal *et al.*, 1986; Armitage *et al.*, 1985). The correlation of DNA polymerase α staining with the DNA ploidy pattern suggests the usefulness of DNA polymerase α positive cells rate in judging the malignancy grade of carcinoma.

The DNA polymerase α positive cells rate was histopathologically examined in large bowel cancer lesions. The results revealed that the ratio of positive cells was increased as the degree of differentiation of cancer decreased. For patients with total invasion of the large bowel wall, the rate of DNA polymerase α positive cells was higher than for those with partial invasion of large bowel wall, and the rate of DNA polymerase α positive cells also correlated with the presence of venous invasion or liver metastasis. In other words, the rate of antibody positivity seemed to allow the rate of proliferating cells to be estimated, thus helping to predict the tendency for invasion and the proliferative potential of the tumours. Although this study was retrospective in nature, the rate of DNA polymerase α positive cells can also be analysed with biopsy specimens. Thus, DNA polymerase α positive cell rate may possibly be a useful prognostic marker for colorectal cancers.

### References

ARMITAGE, N.C., ROBINS, R.A., EVANS, D.F., TURNER, D.R., BALDWIN, R.W. & HARDCASTLE, J.D. (1985). The influence of tumour cell DNA abnormalities on survival in colorectal cancer. *Br. J. Surg.*, **72**, 828.

BARIL, E.F., JENKINS, M.D., BROWN, O.E., LASZLO, J. & MORRIS, H.P. (1973). DNA polymerase I and II in regenerating rat liver and Morris hepatoma. *Cancer Res.*, **33**, 1187.

BARLOGIE, B., RABER, M.N., SCHUMANN, J. & 6 others (1983). Flow cytometry in clinical cancer research. *Cancer Res.*, **43**, 3982.

BENSCH, K.G., TAKABA, S., HU, S.-Z., WANG, T.S.-F. & KORN, D. (1982). Intracellular localization of human DNA polymerase α with monoclonal antibodies. *J. Biol. Chem.*, **257**, 8391.

BURGER, P.C., SHIBATA, T. & KLEIHUSE, P. (1986). The use of the monoclonal antibody Ki-67 in the identification of proliferating cells. *Am. J. Surg. Pathol.*, **9**, 611.

CHANG, L.M.S. & BOLLUM, F.J. (1973). A comparison of associated enzyme activities in various deoxyribonucleic acid polymerases. *J. Biol. Chem.*, **248**, 3398.

GERDES, J., LEMKE, H., BAISCH, H., WACKER, H.-H., SCHWAB, U. & STEIN, H. (1984). Cell cycle analysis of a cell proliferation associated human nuclear antigen defined by the monoclonal antibody Ki-67. *J. Immunol.*, **133**, 1710.

GERDES, J., LELLE, R.J., PICKARTZ, H. & 5 others (1986). Growth fractions in breast cancers determined in situ with monoclonal antibody Ki-67. *J. Clin. Pathol.*, **39**, 977.

GRATZNER, H.G. (1982) Monoclonal antibody to 5-bromo and 5-iodeoxy uridine. A new reagent for detection of DNA replication. *Science*, **248**, 474.

KOKAL, W.A., DUDA, R.B., AZUMI, N. & 4 others (1986). Tumor content in primary and mestatic colorectal carcinoma. *Arch. Surg.*, **121**, 1434.

LELLE, R.J., HEIDENREICH, W., STAUCH, G. & GERDES, G. (1987). The correlation of growth fraction with histologic grading and lymph node status in human mammary carcinoma. *Cancer*, **59**, 83.

LOVETT, E.J., SCHNITZER, B., KEREN, D., FLINT, A., HUDSON, J.L. & MCCLATCHEY, K.D. (1984). Application of flow cytometry to diagnostic pathology. *Lab. Invest.*, **50**, 115.

MASAKI, S., SHIKU, H., KANEDA, T., KOIWAI, O. & YOSHIDO, S. (1982). Production and characterization of monoclonal antibody against 10 S DNA polymerase α from calf thymus. *Nucleic Acids Res.*, **10**, 4703.

MATSUKAGE, A., YAMAGUCHI, M., TANABE, K., NISHIZAWA, M., SETO, M. & TAKAHASHI, T. (1982). Establishment of hybridoma clones which produce anti-chick embryo DNA polymerase α monoclonal antibodies. *Gann*, **73**, 850.

MATSUKAGE, A., YAMAMOTO, S., YAMAGUCHI, M., KUSAKABE, M. & TAKAHASHI, T. (1983). Immunocytochemical localization of chick DNA polymerase α and β. *J. Cell. Phys.*, **117**, 266.

McGUNIN, J.F., DORIA, M.I., DAWSON, P.J., KARRISON, T., STEIN, H.O. & FRANKLIN, W.A. (1987). Assessment of tumor cell kinetics by immunohistochemistry in carcinoma of breast. *Cancer*, **59**, 1744.

MUSHIKA, M., MIWA, T., SUZUKI, Y., HAYASHI, K., MASAKI, S. & KANEDA, T. (1988). Detection of proliferating cells in dysplasia, carcinoma in situ, and invasive carcinoma of the uterine cervix by monoclonal antibody against DNA polymerase α. *Cancer*, **61**, 1182.

NAKAMURA, H., MORITA, T., MASAKI, S. & YOSHIDA, S. (1984) Intracellular localization and metabolism of DNA polymerase α in human cells visualized with monoclonal antibody. *Exp. Cell Res.*, **151**, 123.

SARNGADHARAN, M.G., ROBERT-GUROFF, M. & GALLO, R. (1978). DNA polymerases of normal and neoplastic mammalian cells. *Biochim. Biophys. Acta*, **516**, 419.

SASAKI, K. (1977). Measurement of tritiated thymidine labelling index by incubation *in vitro* of surgically removed cervical cancer. *Gann*, **68**, 307.

SCOTT, N.A., GRANDE, J.P., WEILAND, L.H., PEMBERTON, J.H., BEART, R. & LEIBER, M.M. (1987). Flow cytometric DNA patterns from colorectal cancers – how reproducible are they? *Mayo Clin. Proc.*, **62**, 331.

TANAKA, S., HU, S.-Z., WANG, T.S.-F. & KORN, D. (1982). Preparation and preliminary characterization of monoclonal antibodies against human DNA polymerase α. *J. Biol. Chem.*, **257**, 8386.

VAN DIERENDONCK, J.H., KEIJZER, R., VAN DE VELDE, C.J.H. & CORNELISSE, C.J. (1989). Nuclear distribution of the Ki-67 antigen during the cell cycle: comparison with growth fraction in human breast cancer cells. *Cancer Res.*, **49**, 2999.

YAGURA, T., KOZU, T., SENO, T. & TANAKA, S. (1987). Immunochemical detection of a primase activity related subunit of DNA polymerase α from human and mouse cells using the monoclonal antibody. *Biochemistry*, **26**, 7749.

YAMAGUCHI, A., ISHIDA, T., YABUSHITA, K. & 5 others (1988). The correlation of expressing a nuclear antigen reactive with monoclonal antibody Ki-67 with degree of malignancy in colorectal cancer. *Oncologia*, **21**, 82.

WEISSBACH, A. (1979). The functional roles of mammalian DNA polymerase. *Arch. Biochem. Biophys.*, **198**, 386.

WOLLEY, R.C., SCHREIBER, K., KOSS, L.G., KARAS, M. & SHERMAN, A. (1982). DNA distribution in human colon carcinomas and its relationship to clinical behaviour. *J. Natl Cancer Inst.*, **69**, 15.